# What Do We Know When We Know a Compulsive Buying Person? Looking at Now and Ahead

**DOI:** 10.3390/ijerph191811232

**Published:** 2022-09-07

**Authors:** José Manuel Otero-López

**Affiliations:** Department of Clinical Psychology and Psychobiology, Faculty of Psychology, C/Xosé María Suárez Núñez, s/n, Campus Vida, University of Santiago de Compostela, 15782 Santiago de Compostela, Spain; josemanuel.otero.lopez@usc.es; Tel.: +34-8818-13882

**Keywords:** personality, dispositional traits, personal concerns, characteristic adaptations, life stories, compulsive buying

## Abstract

Over the last few decades, research has seamlessly confirmed the marked multicausal nature of compulsive buying, since variables from different realms (e.g., family, social, and contextual domains) have demonstrated their explanatory capacity. However, it has been personality variables that have, to a greater extent, aroused the interest of researchers, leading to what is now a cumbersome richness of personal constructs of different nature that seem to require some arrangement under integrative frameworks. The proposal by McAdams under the suggestive title of “What do we know when we know a person?” is, in this regard, especially attractive and thought-provoking. McAdams approaches us to the person as a whole by establishing three differentiated levels (dispositional traits, personal concerns or characteristics adaptations, life story), and it will be precisely these levels that will become the structure we will use to address the state of the art on compulsive buyers. The location of the multiple personal variables analyzed at each of the levels with a common grammar will allow us to identify what it is known, as well as what remains to be done in each space. Lastly, suggestions for the future are given, with particular emphasis on advancing our understanding of the person from each of the academic vantage points but also the suitability of designing studies that integrate and/or build bridges between dispositional traits, characteristic adaptations, and life narratives. The hope is that research in the coming years satisfactorily integrates the different visions of the person to achieve a more comprehensive, nuanced portrait of a person with compulsive buying problems.

## 1. Introduction

In the last few decades, buying behavior has been under scientific scrutiny. Indeed, both theorists and researchers have witnessed the emergence and consolidation of a behavioral problem whose leitmotiv is the psychic need and/or the urge to buy [1,2,3]. Compulsive buying, pathological buying, shopping addiction, excessive buying, buying–shopping disorder, and compulsive buying–shopping have been some of the rubrics for a growing phenomenon that currently attracts the interest of many members of the scientific community interested in the field of health.

Compulsive buying is defined by a preoccupation with buying and shopping, by frequent buying episodes or by overpowering urges to buy that are experienced as irresistible and senseless [4]. It often turns into a primary response to negative feelings that provides immediate short-term gratification and even a way of generating or chasing positive mood but which ultimately causes harmful consequences at personal, family, financial, and social levels [1,5,6,7]. In persons with compulsive buying seeking treatment, anxiety, depressive, substance abuse, and personality disorders, as well as other behavioral addictions (e.g., pathological internet use or pathological gambling), are frequent [4]. In the last few years, a great many epidemiological studies have been conducted in different cultural settings and with a variety of samples that seek to account for the scope of the phenomenon [8]. Thus, from samples of the general population [7,9,10,11,12], shopping mall visitors [13], and university students [14,15,16,17,18], prevalence rates of compulsive buying between 3.6% and 18.5% were confirmed.

In this context, and in an attempt to approach the status quo of research, it should be underscored that, in recent years, important research groups have been created in different countries, which, from a variety of disciplines (psychology, sociology, psychiatry, anthropology, marketing…), are working for a common interest: better knowing–understanding–explaining the phenomenon of compulsive buying. Therefore, this theme (compulsive buying) is being the object (or the subject) of multiple studies, which clearly indicates that we are before an expanding field with broad interests. Exciting work is underway. Current research, although it follows disparate paths, seems now more coherent and promising when compared to the past. An overview of the literature shows that family, social, and contextual determinants play a key role in the genesis and continuation of compulsive buying [19,20]. However, it has been personal variables that have, to a greater extent, attracted the interest of researchers. Traits [21], materialistic values [22], self-esteem [23], impulsivity [24], narcissism [25], depression [11], anxiety [26], self-efficacy [27], optimism [7], and coping strategies [28] have, among others, shown to be valid personal predictors of compulsive buying. Given such plethora of determinants from the scenario of personality, integrative frameworks are needed that make it possible to provide order and integrate the different influences, but these frameworks should also provide a “conceptual structure” from which to posit and base future research. 

In this connection, although there are other proposals in the field of psychology of personality for the arrangement of variables [29,30,31], it is the level model put forward by McAdams in 1995 that seems to us particularly suggestive and useful to present and arrange the current state of the art about compulsive buyers. 

The proposal by McAdams [32], although it has been qualified over time [33,34,35,36], was presented under the original title of *What do we know when we know a person?* and it will be the cornerstone and the “buttress” that will guide the development and rationale of this study. Indeed, the different levels that the author posits will provide the structure and the context for accommodating the different personality units that have been developed in the field of compulsive buying so that we may glimpse what it is known in each space. Not least important for the progress in the knowledge about compulsive buyers, also from this model, will be reflecting on what remains to be done at each level of analysis and/or some suggestions for future research. It seems therefore necessary to briefly review the different scenarios or levels that McAdams [32] put forward. On the first level, we find traits. They provide a stable, comparative, and relatively decontextualized sketch of the person. This level locates the person within a series of linear dimensions with proven social meaning. Although this unit has gone through some critical times, in the last few decades, it has recovered its protagonist in the study of the persons thanks to the Model of the Big Five [29]. This model posits the existence of five dimensions: extraversion, neuroticism, agreeableness, conscientiousness, and openness to experience. The Revised NEO-Personality Inventory (NEO-PI-R; [37]), as an assessment tool for the “Five”, has been widely used in the field of clinical psychology and has led to some important findings. For McAdams [32], however, the usefulness of traits lays in that the information they provide is valuable for a first assessment of persons we barely know. The author sees traits as a “psychology of the stranger”, the usefulness of which is a first (and necessary) reading of the person. 

Level II means moving from “having” (traits) to “doing” [38]. Under the generic label of “personal concerns” (or more recently characteristic adaptations), this level accommodates a heterogeneous set of units that are typically coached in motivational, developmental, or strategic terms. We find here, among other potential non-trait variables, such constructs as motives, values, personal strivings, personal projects, current concerns, life tasks, coping strategies, virtues, cognitive schemas, and development tasks. They refer, according to the author, to what people wish during specific periods of their lives and what methods they use (strategies, plans, defenses…) to achieve what they want (and avoid what they do not want). Their contextualization in time, place, and role is the key difference with traits [32]. In other words, behavior cannot be well predicted unless we are able to understand people well, unless we consider how people contextualize their own lives and how their lives are placed in time, place, and role. 

The life story, expressed in narrative terms, makes up level III. A life story is, according to McAdams [32], a narration of the internalized, evolving self with a sense of unity and purpose, which adds the reconstructed past, the perceived present, and the anticipated future: the person weaves a self-defining narrative account, arranged in a time sequence full of scenes, characters, plots, and themes. This makes up the “material” that this person has been collecting throughout his or her life, which integrates and gives coherence to his/her life (roles, values, skills, challenges…). Beyond dispositional traits (the “having” of level I) and contextualized personal concerns (the “doing” of level II), the “construction of the self” (the identity) would emerge from this narrative lens of the third level. 

Ultimately, the author suggests that, at the first level, is the stranger; at the second is the individual who acts and makes an effort in specific scenarios of their life; at the third, the person finds unity, coherence, meaning, and purpose in life. Besides, from this three-level framework of study of personality, McAdams [34] holds that people may be understood from three different psychological points of view: as actors, agents, and authors, so that the first level corresponds to the self as a social actor. Beyond this level is the self as a motivated agent, and lastly is the autobiographical author, “the self-as a storyteller who ultimately aims to burnish and synthesize episodic information about the self into a coherent and integrative life story” ([34], p. 273).

Therefore, once familiar with the academic vantage points from which a person can be studied, there only remains to ask ourselves (and this is precisely the starting point of this work), *what do we know when we know a compulsive buying person?* and, derived from this question, *how can we know a compulsive buying person better?* taking into account the findings of the productive lines of work that crowd the field. It is not solely about providing order to what it is known about the person with compulsive buying problems within McAdams’ markedly integrative model but also about sketching some lines from this conceptual framework that should guide future research to achieve a more comprehensive, nuanced portrait of a person with buying problems. 

## 2. Level I—Dispositional Traits and Compulsive Buying

At level I, and despite the fact that work has been conducted on isolated traits such as impulsivity [24,39], sensation-seeking [40], or narcissism [25,41], for some years now, it has been the Big Five Model [29,37] that has had the highest heuristic value with regard to compulsive buying. An overview of the previous literature shows that there are differential association patterns with compulsive buying on the basis of the domain analyzed. 

Thus, extraversion (the tendency to be sociable, warm, active, assertive, cheerful, and in search of stimulation) has been confirmed in a number of studies [42,43,44,45,46,47] as having significant positive relationships with compulsive buying. The need to search for stimulation that characterizes an extrovert person could at least partially explain this finding. However, some studies [48,49,50,51,52] also show that the association between extraversion and compulsive buying does not reach statistical significance.

As to agreeableness (the dimension of interpersonal relations, characterized by altruism, trust, modesty, and cooperativeness), results are contradictory. While some studies report a positive association with compulsive buying [46,53,54], others confirm a negative relation [42,43,44,47,48,50,51,52]. Tentative hypotheses could be posited to account for both types of findings. People with high scores in agreeableness, given their propensity to trust the intentions and purposes of others, are probably more vulnerable to marketing strategies and pitches, thus increasing the probability of compulsive buying. On the other hand, it could be argued that more agreeable persons, as people who avoid interpersonal conflicts, are less likely to get involved in this problem, as it usually leads to serious problems in their relationships with other people close to them. 

Research on the relation between openness to experience (the tendency to be imaginative, creative, unconventional, emotionally, and artistically sensitive) and compulsive buying yields contradictory results. Some authors report positive relations [46,47], while other authors confirm a negative relation [42,43,44,48,49,52,53], and there are also studies [45,51] wherein the relation does not reach statistical significance. Explanatory hypotheses could be suggested for each pattern of findings. Indeed, while the buying experience could satisfy the curiosity and seeking of originality that characterizes those who score high in this personal dimension, it would also be plausible to argue that it is precisely their non-conventional ideas and values that drive them to seek and engage in other, different activities that do not involve buying.

A completely different picture emerges as far as the robustness of the findings with respect to the relation between neuroticism (the tendency to experience negative emotions, such as anxiety and depression) and conscientiousness (the tendency to be organized, strong-willed, persistent, reliable, and a follower of rules and ethical principles) and compulsive buying. An overwhelming majority of studies show a positive and significant relation with regard to neuroticism and a negative relation in the case of conscientiousness and compulsive buying [42,43,47,48,49,50,51,52,54,55]. Compulsive buying often becomes a way out of negative emotions, which seriously compromises self-control.

As well as the study on the broad dimensions of the Five-Factor Model, there is a recent study [56] that also analyzes the facets (an aspect that had not been previously explored in this field of work). The results demonstrate that subjects with high propensity to compulsive buying present the highest significant levels in neuroticism and the lowest levels in conscientiousness and agreeableness. Besides, all of the facets of neuroticism and conscientiousness and most of the agreeableness facets (namely, straightforwardness, altruism, trust, and modesty) establish significant differences between groups. A most interesting finding is that, even when the domain of extroversion and openness did not establish significant differences on the basis of vulnerability to compulsive buying, statistically significant differences do actually appear when looking at some of their facets, specifically, the facets of excitement-seeking, positive emotions, and assertiveness, corresponding to the domain of extraversion and aesthetics and ideas for the domain of openness to experience.

Another research path that belongs in this first level of analysis of compulsive buying people is that which has tried to identify personality prototypes based on the Big Five. In an interesting study [57], two distinct personality prototypes in treatment-seeking patients with compulsive buying were identified through cluster analysis. Specifically, subjects in cluster II scored significantly higher than those in cluster I on neuroticism and lower on extraversion, agreeableness, openness, and conscientiousness. 

A final, extremely interesting line of work that has undoubtedly contributed to shedding light on the dynamics of influence between the Big Five and compulsive buying is the one positing a variety of causal approaches. The models proposed generally consider the Big Five as exogenous variables, a wide range of variables with a potentially mediating role and compulsive buying as the endogenous variable. Specifically, and in order to summarize the main proposals, attention has been paid to whether materialism [50,54], impulsive buying [58], gluttony [59], hedonistic shopping experiences [47], negative perfectionism [55], or a past-negative time perspective [60] channel the influence of the five in compulsive buying. From the main results obtained in the different studies, it has been confirmed that conscientiousness has a direct negative effect on compulsive buying [47,50,54], while the positive impact of extraversion on this problem is mediated by materialism [50,54] or hedonistic shopping experiences [47]. Furthermore, a positive indirect effect of openness to experience is confirmed for compulsive buying through materialism [54], impulsive buying [58], and hedonistic shopping experiences [47]. As to neuroticism, evidence is found of both direct positive effects [50,54] and indirect positive effects through materialism [50,54], impulsive buying [58], gluttony [59], hedonistic shopping experiences [47], perfectionism [55], and a past-negative time perspective [60] in compulsive buying.

In short, an overview of studies on compulsive buying on the “dispositional traits” (level I of McAdams’ proposal) confirms that the Big Five have a notable heuristic value. Specifically, of the Big Five of personality, neuroticism emerges as the most important factor implicated in compulsive buying. There is outstanding evidence of its positive and significant covariation with this behavioral problem (its influences on the causal models reviewed are both direct and indirect). Conscientiousness appears to be, besides neuroticism, another extremely relevant Big Five personality factor for the understanding of compulsive buying (direct and negative effects in the different causal approaches). The role of the other three Big Five factors of personality in compulsive buying is less clear. At a correlational level, results have little consistency. Some studies find statistically significant relationships—positive or negative—while other studies find that the relationship lacks statistical significance. On the face of these contradictory results, we hold that further research is needed that contributes to the consolidation of empirical evidence and clarifies some unsolved issues. In this regard, it is particularly interesting for progress in this area of knowledge, as it entails a finer analysis than that provided by dimensions, to further study the facets (there is only one single study in the field, and its results clearly suggest that facets may capture unique personality information, but the results are overshadowed as the study then focuses exclusively on broad factors). Another pending challenge, as we have noted there is only one study, and which, in our view, may be both stimulating and productive for the field, is the search for personality styles (specific combinations of traits) from the Five Factor Model in compulsive buyers. Previous studies conducted in neighboring fields such as tobacco consumption [61] or binge eating and drinking [62] support this line of work. Ascertaining what combinations of traits increase the risk of compulsive buying clearly seems to be necessary to design any action proposal. Lastly, the possibility of relating the Big Five with variables or constructs from the remaining levels (personal concerns and life story) would be another avenue for future research. 

## 3. Level II—Personal Concerns, Characteristic Adaptations, and Compulsive Buying

As well as taking into consideration the dispositional traits of level I (the individual as a social actor), the McAdams model [32] puts forward a second level that includes several facets that make up human individuality (goals, motives, values, plans…) contextualized in time, place, and role (the person as a motivated agent). 

Specifically, a number of personality constructs have been analyzed. Materialism, conceptualized as a personal value [63,64], has generated considerable interest among scholars. The extensive literature on this issue confirms that regardless of the type of sample under study, materialism is positively associated with compulsive consumption [65]. By way of example, the study by Dittmar [22] is illuminating, as materialism is found to be the strongest predictor of individuals’ compulsive buying from three samples (adults who had contacted a self-help organization, younger adults from a multinational corporation’s consumer panel, and adolescents). Similarly, materialist values emerge as a significant predictive variable of compulsive buying in other studies conducted with university students [18,66,67], adolescents [68], the general population [9,69], and clinical samples [70]. 

In the last decade, based on the empirical confirmation of the strong link between materialism and compulsive buying, many studies have sought to clarify the mediating role of other variables. It has been demonstrated that variables such as self-esteem [71], life satisfaction [72], hedonic values [73], time affluence [74], anxiety and depression [75], money management [49], irrational buying-related cognitions [76], and hedonistic shopping values [77] channel the influence of materialism on compulsive buying. 

As well as materialism, some authors have looked at the effect of other values and goals on compulsive buying. Specifically, and on the basis of the classification of values made in the personal values theory [78], the positive association of stimulation and hedonism with compulsive buying has been confirmed [79,80], as well as the protective effect of self-transcendence and conservation values in the development of compulsive buying [79]. Research has shown that people who are characterized by a reciprocal and egoistic value orientation show high vulnerability to compulsive buying, while prosocial value orientation acts as a protective factor for this behavior [81].

The study of personal goals or life aspirations [82] developed from the framework of the self-determination theory [83,84] has also been an important topic of research. In this line, the work by Roberts and Pirog [85] is particularly relevant: using a sample of university students, a positive association was confirmed between compulsive buying and extrinsic goals in financial success and attractiveness and a negative association with the intrinsic goals of self-acceptance and community feeling. Empirical support to the role played by this motivational unit is also found in other studies conducted using both general population samples [51,86] and university students [87]. Specifically, Otero-López and Villardefrancos [86] conclude that it is the people who are more vulnerable to compulsive buying that have the highest scores in the importance given to all extrinsic goals and in the probability of achieving extrinsic image and conformity goals. They also report the lowest levels in the probability of achieving intrinsic goals. In a later study, these authors [51] conclude that the extrinsic goals of image, popularity, and conformity strengthen the predictive power of personality traits in compulsive buying; more recently, Otero-López, and Santiago and Castro [87], confirmed that the life aspirations of image, popularity, and hedonism act as risk factors in compulsive buying, while the importance granted to the intrinsic goals of self-acceptance and affiliation operate as protective factors. 

Similarly, and in line with the growing interest that the so-called personalized goals [88] have aroused in the last few decades, the appraisal of personal projects, i.e., the extended sets of personally salient actions in contexts that range from the daily affairs to the self-defining passions of the lifetime [89], has been the object of analysis in a recent study [90]. The results obtained allow us to conclude that it is university students with high vulnerability to compulsive buying who appraise their personal projects as more stressing and less significant and structured, and, besides, they see themselves as less efficient to cope with them. In particular, it should be noted that, on the basis of the dimension of the self-identity of the broad domain of meaning, the low scores by subjects that are more vulnerable to compulsive buying seem to reflect a prototypical characteristic of compulsive buyers: engaging in compulsive buying as a way of compensating and or restoring identity.

Within this level where a person is considered a motivated agent (what people strive for, value, believe about how well they are doing in that domain), research has been conducted, although with varying degrees of attention to the link of other constructs (self-esteem, self-efficacy, coping strategies) to compulsive buying. 

Self-esteem has been for decades one of the variables that has best characterized compulsive buyers [91,92]. Looking back, the pioneering work of O’Guinn and Faber [93] stands out. Starting with a study that combines quantitative and qualitative methodology, they found that compulsive buyers not only score significantly lower in self-esteem, but also, in their narrative accounts, they continuously mention feelings of little worth as persons. The abundant literature around this personal construct has confirmed its relevance in explaining and predicting this problem [23,69,71,94,95]. In recent years, several researchers have added self-esteem, along with other variables, to their studies. For instance, Villardefrancos and Otero-López [18] underscored that, among students, compulsive buyers, when compared to non-compulsive buyers, obtained significantly lower levels in self-esteem, life-satisfaction, and optimism. Biolcati [96] concludes, in a sample of women, that low self-esteem is a significant predictor of compulsive buying, this relationship being partially mediated by the fear of being negatively appraised by the others. Other studies show that family conflict had a significant indirect effect on compulsive buying through self-esteem [68] and that the effect of perceived stress on online compulsive buying is moderated by self-esteem [97]. Lastly, a very recent study [98] shows that the relationship between positive and negative self-esteem and online impulsive buying is significantly weaker than that of self-esteem and online compulsive buying.

Self-efficacy has aroused the interest of some very recent studies [16,27,52,99,100] that confirm that compulsive buyers show low self-efficacy.

The study of the strategies that characterize the way persons vulnerable to compulsive buying cope with stress has been the subject of some studies conducted in the last decade [7,28,101]. These studies conclude that mental disengagement [28] and strategies of problem avoidance, wishful thinking, and self-criticism are risk factors for compulsive buying, while problem solving, cognitive restructuring, and social support are protection factors [7,101]. There have also been studies wherein coping has been considered the mediating variable. It has been confirmed that negative coping mediated the association between the perception of stress and online compulsive buying [97] and that the helpless coping style channels the anxiety felt due to COVID-19 and compulsive buying [102].

Lastly, generativity, or the concern and activity devoted to contributing to others and society [103,104], although it has been linked to sustainable and/or responsible consumption [105], it has received little attention in the field of compulsive buying. There is only one study [87], that concludes that generativity, along with other variables, such as the intrinsic goals of self-acceptance and affiliation, is a protective factor against compulsive buying. 

In sum, in the field of compulsive buying, significant progress has been made with regard to a domain of human individuality that is closely linked to motivation and cognition. Research has confirmed the suitability of different variables (materialism, self-esteem, values, and goals) to better explain and understand the behavior of a person with buying problems. It is also true, however, that given the wide range of units susceptible of being included in this second level, much work remains to be done. Some suggestions: (1) personalize the motivation of the compulsive buyer from an analysis of units or “personal action constructs” (personal projects, personal strivings, current concerns, life tasks…) that help know what they pursue and how they give meaning to their lives; (2) clarify what strategies and tactics people with compulsive buying use to cope with and manage their reality; (3) add new personal constructs (generativity, for instance) to the study of compulsive buying; and (4) clarify how the “doing”, loaded with purpose and intent, channel or modulate the influence of the “having” on compulsive buying. This last aspect is particularly important not only to build bridges between the different levels (the notion underlying this study) but also to provide an opportunity to get to know better the dynamics, interactions, and synergies that bring us closer to a better and fuller understanding of the compulsive buying person. We believe that these aspects, among others, should be part of an agenda for the future. 

## 4. Level III—Life Story and Compulsive Buying

The construction of identity as an objective, the life story being the vehicle for narrative, characterizes this level of analysis of the person. This level, according to McAdams [34], looks at the individual as an autobiographical author and suggests that the life story creates meaning and purpose in his/her life by constructing self-defining stories. 

Despite the fact that in the field of personality, there is an important tradition in the use of idiographic methods for the study of individuality (case studies, psychobiographies, life stories), very few researchers paid attention to the life of a compulsive buyer. 

The overview of the literature confirms the existence of a variety of studies that, using narration, illuminate the phenomenological description of compulsive buying and delve into the life story of compulsive buyers. A pioneering work in the field is O’Guinn and Faber [93] who interviewed compulsive buyers about a variety of issues (first realization of the problem, its course, its relation to significant life events, and a detailed description of a buying episode). The search for common answers and themes by compulsive buyers confirms, among other things, that feeling guilty, dissatisfied with oneself or unattractive are common expressions found in the narrative accounts made by these persons. In this regard, and in connection with the self-assessment domain, a person with a compulsive buying problem told the following:

*“I have a brother who is now a dentist, who was everything Mother and Dad ever wanted without question. He was bright and he was very engaging, and he is very well to do and all of that. And then there is (informant’s name) and my mother did my school work ever since I was in fifth grade. She did all of my school work even my college papers. It’s not much to be proud of”*.

[93] (p. 153)

Scherhorn, Reisch, and Raab [106] conducted in-depth interviews with an extreme group of self-identified addictive buyers, taking an interest in key aspects of their life trajectory throughout the different developmental stages (childhood, adolescence, and adulthood) considering the buyer´s individual socialization and the societal conditions under which the buying behavior originated. The fundamental conclusion of this study is that buyers, from the perception that, for other significant persons (parents, close relatives, neighbors) material goods seemed to be more important to them than themselves, have learnt to compensate for their lack of self-esteem by buying material goods. 

Other researchers [107,108], using phenomenological interviews, provide compulsive buyers with the opportunity to tell their own story and discuss both aspects of their behavior and their lives. In particular, Eccles [107] explored the personal stories of female addictive consumers to get to know how and why this behavior developed to the point of becoming the center of their life. The author confirms, from the analysis of the content of the interviews, the existence of different patterns or subgroups of addictive consumers (existential addict, the revenge addict, the mood repair addict, and the serial addict), motivation being the main factor of the lack of homogeneity in this problem. By way of example, it is very enlightening to read the account of a person about the impact that her marriage relationship has had on the development of addictive consumption:

*“He loves to work (referring to her husband) and, in the early days when the children were very small and I was bringing them up practically single-handed, he would work Saturdays and Sundays. I would resent that so I would think, “Right, if he’s working, then I’m spending.” Now we’re in a vicious circle—I’m spending and he’s working. And I say, “Can’t you cut down the work? Can’t we go away for the weekend?” And he says, “How can I? I have to work to pay the bills.” In the early years we would have big rows about it”*.

[107] (p. 13)

The thematic analysis of the journal of an addictive buyer is the objective of García [109]. This journal is a great material to gain an in-depth understanding of addictive buying and the theories on the causes behind the behavior of the female narrator. The distant causes (in which the family plays a key role), together with the immediate or triggering causes of the addictive behavior linked to life-changing situations (maternity and marriage crisis), account for the appearance and evolution of this addictive buying problem. Specifically in connection to the family as a cause for her problem, the author of this diary explains:

*“I ask myself now whether my problem is inherited, or whether it’s what I’ve seen, or whether I’m a dissolute person. Because my older brothers aren’t like that. The women are, though, they lied about shopping, they always said it cost less than it had; well, not when buying food; then, they said it had cost a bit more… My family, a total disaster. I don’t know to what extent this might have been an influence, a lot, I think”*.

[109] (p. 416)

The interview format of the life story proposed by McAdams [32] has only been applied, to our knowledge, in a single research project [2,110], the fundamental objective of which was to gain an understanding of how a compulsive buyer (fictitious name “She”) had been building her identity from her life events. Thus, throughout the interview, the narrator identifies the main vicissitudes that have characterized her life trajectory, how she has organized her past, perceives her present, and anticipates her future; which themes have been most present throughout her life; buying as a core element of her story; and, in short, how all this gives meaning and purpose to her experience as a person. 

Since, as noted, this is the only study conducted with a compulsive buyer following the interview protocol proposed by the author of the model that underpins this work, we include below some aspects taken from her narrative account that allow us to approach the way She has been building her identity [2]. 

The protagonist divides her life story into three chapters, each of them with its own title: chapter 1, “Untroubled childhood and discomfort at school”; chapter 2, “Adolescence broken by the separation of my parents”; chapter 3, “Living alone, making money and buying”. As to this third chapter (the beginning of her problem), she says:

*“I would sum up my most recent years, and I do think this is a different part of my life, from the moment I left home. I was ina bad place, sick and tired of everything and looking forward to being my own person… for me it was very important to find freedom and have a job. My money gives me the possibility to decide. Well, sometimes it leads you down an undesired path, just look at me, for example. For me, money meant that I could buy many things that I had always wanted to buy. I have always liked to look good. For me, physical appearance, what others see me like, is important in life. I bought handbags, purses, I have always liked those things and a lot of clothes at the beginning of all this (she means her current buying problem), and I was very happy with myself: it was my money and I spent it on what I wanted. Well, then things changed, little by little, you get hooked and what you buy, which at first made you feel very well, then you realize you have gone too far. You feel good when you buy, but then you blame yourself and often feel bad for having spent the money”*.

(p. 40)

After organizing her life story in several chapters, the protagonist identifies seven key scenes and episodes that she judged to be highly significant in her life (see Table 1), which allow us to somehow know who she was and who she is a person. 

It is especially interesting to note that, in the scenes from her past, the recollection of experiences with a high negative load (e.g., the divorce of her parents or her break-up with her first partners) intermingle with times of personal independence in which buying takes on special prominence. Other important elements should be also mentioned in the analysis of her life story: an optimist narrative tone and a determined desire for self-improvement (“…right now I try to be happy, focusing on my partner, my home, having a job and especially a quiet life…”). As to the thematic lines of the story, it is peppered with both agency themes (independence, self-expansion) and communion (intimacy, love, attachment), which in some parts of her narrative account seem to open a window into some displacement and change (“…now I am focusing more on people rather than on things because now I realize that back then I lived and worked to have more and more things and in the end I did not enjoy anything”). As to the theme, message, or core idea of her life story, the narrator responds:

*“The theme of my story? Actually, I don’t know. I think I can be up and suddenly completely down. Sometimes good things come your way. Sometimes bad things. Something like that. Sometimes life makes things hard for me and then I look and despite how bad things are, which seem really very bad, there is always a way out. Well, in my case there is not a theme, there are several. I am here because of the buying, there are also my failures. In short, I don’t know. Sometimes I feel like I was in a roller coaster or playing roulette; that would be the theme …”*.

(p. 49)

In sum, the interpersonal conflicts in the life trajectory of “She”, which seem to have left an important and indelible mark, explain, to a great extent, her attempts to restore her emotional state and improve her self-image from owning certain material goods. Buying, as she explains, worked (albeit temporarily) as an antidote to her emotional distress:

*“It used to happen to me when I had some problem or concern. Often, when I felt bad, anxious, depressed, my way out of it was buying. While I was trying clothes on, and I was focusing my attention on whether they look good on me I could not think of anything else. Then, at that tiny instant it was like being in other world and I forgot that it had been a bad day, that I had had an argument or that something was happening. It is always a bit like that: you feel bad, and you need to buy”*.

(p. 66)

In short, from the review of the literature, it can be seen that there are very few studies that using narrative methods have sought to look into the life stories of compulsive buyers. The need of this level to capture the integration of the person (looking at the world of meanings, making sense of the experience, the unity, the purposes…) is both urgent and necessary. Otherwise, we may bring back long-gone practices in the study of persons and look at the “parts” to the detriment of the “whole”. Research into compulsive buying should also encourage methodologies and approaches [111,112,113] that make it possible to collect and analyze the life stories of compulsive buyers to complete our knowledge of persons. The collection of life stories from compulsive buyers with different backgrounds (general population, clinical population) with different levels of chronicity, different cultures, different genders, and ages will allow us to transcend the idiographic to identify patterns of common and diverging characteristics in the stories. It will be an important asset for this level of analysis in particular and generally for the field of the study of compulsive buying.

Ultimately, we need traits, and while personal concerns should not be lost sight of, we must not forget identity (the life story as a narrative vehicle that makes it possible to integrate the past-present-future) if we wish to have a full, comprehensive approach to the compulsive buying person. At this point, there only remains to reconcile the findings from the three scenarios (traits, personal concerns, and life history) to be able to sketch a prototypical profile of a person with compulsive buying problems. High neuroticism, low conscientiousness, marked materialism (the importance of having), the prominence of extrinsic goals (particularly, image and popularity) to the detriment of intrinsic goals (especially, self-acceptance and affiliation), a stress-coping style wherein the passive/avoidant strategies tend to prevail (e.g., problem avoidance and wishful thinking) as opposed to other more active strategies (e.g., problem solving, cognitive restructuring, and social support), and a major undermining of self-esteem are some of the features identified by research. The appraisal of personal projects as highly stressing and with low meaning, structure, and efficacy, as well as a greater “self-focus” to the detriment of interest in and/or concern towards other people (low generativity), also seem to characterize, in view of recent findings, persons with compulsive buying. Their life stories seem to be tinged with a negative emotional tone, a recurring need throughout their life trajectory for other persons as figures of attachment and/or providers of support, as a result of different contamination sequences (good or emotionally positive events or circumstances that, with the passing of time, become bad or negative) and as a consequence of the presence of unresolved tensions and conflicts. In other words, if we wish to get to know well a person with compulsive buying problems, we need to coherently address and integrate into a whole the most stable aspects (traits) and the most dynamic ones (personal concerns and life story), the nomothetic perspective (individual differences) without losing sight of the idiographic and the temporality that characterizes their lives (the effects of the past channeled through memories and constructed stories, the goals and expectations that emerge from their view of the future, and the present).

One of the main pending challenges for future research is to build bridges that span the gap between life stories, goals, and dispositions. Any action, whether at a preventive or intervention level, should benefit from that knowledge. 

## 5. Looking Ahead: How Can We Know a Compulsive Buying Person Better?

A overview of the literature in the field of compulsive buying has shown that there are many personal variables, different in nature, that compete to explain this behavioral problem. The need for order, integration, and coherence before this cumbersome wealth of determinants led us to opt for the model proposed by McAdams [32], as it is a particularly useful explanatory framework to respond to the question *what do we know when we know a compulsive buyer?* The dispositional and motivational/purposeful elements, as well as the most personological elements (identity), have become—following the approach posed by the above model—different spaces in which we have placed the different personal variables analyzed in this field of study. From this knowledge of what has been done and what remains to be done (field need), there only remains to draw some avenues for the future that may, to some extent, contribute to shedding light on the question of what to do to learn more about compulsive buyers and this is the very purpose of this section. 

We sketch out next some points of reference that we believe may help articulate our proposal for future research:

(1) *The McAdams model: the proposal we defend.* The field of compulsive buying is currently characterized, as we have already pointed out, by an abundance of studies, scattered findings, and the great variety of personal variables that are analyzed. As a consequence, ordering this complex network of influences seems to be not only necessary but also urgent. Although there are other models in the domain of personality [29], we have based our proposal on the McAdams model [32], as it is a most suggestive conceptual framework and one that is extremely useful both for identifying and classifying what it is known about the compulsive buying person and guiding future research.
(2) *Towards the “comprehensive understanding” of the compulsive buying person*. Our proposal strives to facilitate the coexistence of different aspects (the broad and the specific, what is most endogenous and what is most situational, the statical and the dynamic elements) that need to be looked at to understand the complexity of the person and their behavior (compulsive buying, in this case). In other words, we believe that future research, while paying due attention to the study of isolated variables, should also allow for a healthy, integrative, and inclusive approach that brings together the different units that have emerged in the field to fully apprehend the compulsive buying person “as a whole”. Individual differences (level I), intentions or purposes (level II), and life story (level III) are, in our view, domains that need to be explored in depth. The purpose is therefore to complete the study of personal variables from a variety of analytical fronts without seeking complementarity and often using different grammars.
(3) *Building bridges, linking levels.* One of the main hindrances in the field of compulsive buying is the scarcity of works that link the personal units of different levels. Despite the fact that McAdams, in the initial proposal of his model [32], held that the levels on personality should not necessarily be related to one another, later McAdams and Olson [35] claimed, in relation to personality development over the life course, that “It is expected, nonetheless, that dispositional trait, characteristic adaptations, and narrative identity should relate to each other in complex meaningful, and perhaps predictable ways; for after all, this is all about the developmental of a whole person “(p. 530). Fortunately, in the last decades, in the field of psychology of personality, things seem to be changing and notable efforts have been made to link the different levels [114,115,116,117,118]. In this line, our proposal for future research in the field of compulsive buying also suggests this desirable and much longed-for integrative approach. Linking dispositional factors with intentions and purposes, without losing sight of the present–past–future dynamic that drives the life story will allow us to approach from different avenues what makes a compulsive buyer and how they work. Ordering and sequencing the influences (exogenous, mediating…); paying attention to what is shared (individual differences) without losing sight of one’s own; exploring methodological options that have not been tested in the field of study that make it possible to combine quantitative and qualitative elements (mixed methods, for instance) would be some of the pending tasks. In sum, our intention is to convey the notion that future research should, to some extent, echo Murray and Kluchohn [119] that every person is like all other persons, like some other persons, and like no other person, as the objective is to include all things that “add up” and bring us close to a better understanding of the compulsive buying person.
(4) *Paying attention to a high-risk group: the young.* The results of previous research confirming that young people seem to be prone to compulsive buying [8,12,120] establish the basis of the need defended by some authors [18,68,121,122] to design studies aimed at clarifying the scope and variables involved in the compulsive buying behavior of this risk group. In this regard, given the high probability that, at this age bracket, the phenomenon is at its initial stages, knowing what personal dimensions are involved in young-people compulsive buying will provide an opportunity to design early actions with some assurances of efficacy, thus stopping its progress. More specifically, the design and implementation of prevention programs focused on the critical analysis of how certain marketing campaigns contribute to “the creation of needs” and on reflecting on the socio-environmental repercussion of compulsive buying could prove useful to stop the involvement of the young in this problem. Encouraging intrinsic goals to reduce the orientation toward materialistic goals, as defended in some programs [123], seems to be part of the solution.
(5) *Gender and culture: influences that must not be left out*. While there is empirical evidence confirming that women are highly vulnerable to compulsive buying [12,22,106], there is no shortage of studies that, using samples of young people, have failed to find any gender differences [124,125]. Therefore, the important change in the roles of women in current society, the type of items that are bought, the new online buying modes, and even the study of gender differences in pathways to compulsive buying are factors that must be considered for future research in order to delineate the true impact of the gender variable. Shedding light on to what extent culture has an impact on the personality of compulsive buying (and vice versa) and assessing the cross-cultural differences may be a new horizon for future research in this field.
(6) *“Identified” compulsive buyers: progressing in understanding the person.* We also suggest a greater presence of samples of persons with compulsive buying problems in research, as this would undoubtedly provide an opportunity to gain a deeper and better understanding of the dynamics (evolutions, plateaus, and regressions…) that have accompanied the development of this problem. It will also become a fruitful meeting point for all three sources of knowledge of the person: “being–doing–having”. Only from this knowledge and from the analysis of the three levels in these persons (traits–concerns–life history) will it be possible to progress in the understanding of the compulsive buyer.

**Figure 1:**
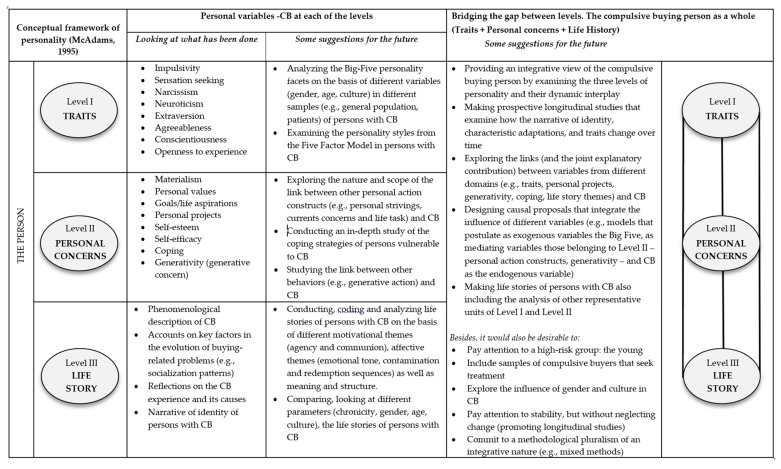
Evidence available with regard to the personal variables and compulsive buying in the light of the conceptual framework of personality by McAdams (1995) along with some integrative suggestions for the future in connection to the different levels of analysis (traits, personal concerns, life story) to study a person who is a compulsive buyer.

In essence and in line with the above (see Figure 1), we would like to reiterate that our proposal for the future seeks to identify some of the potential needs or shortcomings that may be found in the field of compulsive buying with the ultimate purpose of suggesting some potential avenues that may result in accumulative knowledge on this problem. The overview of the previous literature in the field has led us to identify promising strengths, needs, and lines of action. The objective is both to look into whether some tentative findings on which little research has been conducted are confirmed and open new research avenues. We, therefore, put forward some suggestions and possibilities. 

As everything seems to suggest that, in the next few decades, “the Five” will remain the protagonists of level I and will remain to be an important part of personality research, exploring their role (without neglecting the facets) and clarifying whether there are styles that make it possible to evaluate the potential effects of the combinations of these traits will continue to be a requisite if we wish to understand the compulsive buying person. Exploring the links between the personal variables at different levels (traits, personal concerns, life story) and compulsive buying is the second suggestion. Specifically, what it is proposed is not just shedding light on the joint explanatory contribution of variables from different levels (for example, traits, personal projects, generativity, coping…) but also designing causal proposals that integrate these influences. By way of example, we could mention, in the design and testing of causal models wherein the Big Five (or some of them) are exogenous variables, level II units (such as projects, strivings, generativity …) act as mediators and compulsive buying as endogenous variables. Making, coding, and analyzing the life story of compulsive buyers on the basis of motivational themes (agency and communion) and affective (emotional tone, contamination, and redemption sequences) meaning and structure would also be a fruitful addition to progress in the understanding of the phenomenon under study. 

In short, organizing multiple forms of studying the personality of compulsive buyers, adding, and linking units from different levels of analysis, studying samples with special vulnerability to compulsive buying (the young), recruiting compulsive buyers that seek treatment are just some lines that we include in our proposal. Besides, recent studies [102,126,127] that have looked at the current COVID-19 situation confirm that compulsive buying has experienced an increase. Another pending task should therefore be to gain insight into the effects this pandemic has had on the onset, development, and exacerbation of compulsive buying. The making of a life story, this inquiry into how the person (the compulsive buyer) tells and connects their self-defining story to reflect their lives (and thus bringing us closer to their life reality by providing adequate responses to such questions as who is he or she today? How has he or she become who they are today? Where is he or she heading to? ...), extracting the idiographic (one’s own) whilst comparing stories seeking patterns (sliding into the nomothetic) are undoubtedly many additional goals for this future agenda that does not avoid the desirable federation between the qualitative and the qualitative. Being able to look at compulsive buyers from different scenarios, gaining insight into their lives on the basis of what we are looking for (traits, concerns, identity) and facing new ways of grasping and exploring the personal variables of compulsive buyers will probably be the gains of this. Special mention is deserved for the notion of building bridges spanning the different levels (domains) put forward by McAdams [32,34], in the understanding that the person (the personality variables) is a unified whole and that paying attention to just one of the parts (those with a shared grammar) entails major sacrifices (the meaning for the method, for instance). Although McAdams holds in a variety of writings that “there are no reasons to expect strong symmetry and consistency across the different levels of personality description for people’s lives are typically complex and often contradictory” [35], we defend that the “stillness” of the trait should most likely be supplemented by the “intention” and also by the “life purpose” to better capture the essence of the person with compulsive buying problems. But there is more. The findings will most probably have the potential, or at least this is our hope, to better place the “targets” on which to act to reduce the growth of compulsive buying among the young and, not less important, to know which “topics” in the life stories accompany the appearance, development, and chronification of this problem. Most likely, the greater assurance obtained from the knowledge yielded by all three levels will prove helpful for both the person suffering from buying-related problems and the person trying to identify different flanks from which to address the complex variety of cases involving this problem from a preventive or intervention approach. It is our hope that this may be so. 

## Figures and Tables

**Table 1:** Nuclear episodes in the life story of “She” and fragments of the life story.

Nuclear Episode	Fragments of the Life Story
High point: “Having found a partner who had his feet on the ground”	*“The day I broke up with my partner and then when I found another person with his feet on the ground that helps me control myself as much as he can, because of the problem linked to buying… those days in which it seemed I had woken up from a bad dream … being able to say Stop! …When I decided… when I took a weight off my mind, I felt, I don’t know., a great relief”*
Low point: “My parents’ separation”	*“I went out to dinner with my parents and at the table, out of the blue, he tells us he is going to ask for a divorce and that he had another partner. I was astonished I thought so many things ... What was my mother going to do now? … That week was terrifying because it was all threats, thumping of the walls and slamming doors shut. Then I didn’t want to go home… there was an overwhelming sensation of I don’t know … powerlessness … That was unbearable”*
Turning point: “The day I decide to leave my family home”	*“Leaving behind all that shouting, that bad blood, that being always on edge...It was being in hell and then getting out and setting myself free. I have always believed that resentment is a heavy load, and I didn’t want to continue like that. Resentment always takes the place of bitterness and I prefer to be more cheerful, think of other things… I told myself that from that moment on the person I would be with would not impose themselves on me or repress me. Never again. I needed to be myself... To me, being independent came first, the most important thing I had done in my life… I would not let anyone do what they had done to me. Never again. Then, that meant for me saying: Now I am in charge!... I left, I started again… I changed radically. I rented a flat, and well then came the bad stuff with the buying …”*
First recollection: “One morning of the Three Kings Day”	*“I remember being hidden behind the sofa with my bother for quite some time, whispering and we were dying for opening the presents. I particularly remember that desire for the day to come so that I could open the presents…”*
Scene from childhood: “Arguments with my mother about clothes and hairstyles”	*“I especially remember the rows with my mother because of the hairstyles and the clothes she insisted on me wearing … I remember when I went to my uncle’s wedding. No one knows how much I cried and acted up not to wear those clothes. I cried till the day of the wedding. To me it looked horrendous. Actually, I thought about getting rid of them, I had the scissors in my hand, but then I did not dare …”*
Scene from adolescence: “Going to the high school”	*“I often remember that for a time I hung out with a group of girls from another high school who I think instilled in me the excessive concern about dressing well… although that was there before. They were people who went like “I look super good today... I am stunning today, I look super great”, they were very superficial.”*
Scene from adulthood: “When I went to live on my own”	*“I wanted to show the world that I could live alone. I told myself. I am independent. I have a job. I can live in my own flat. It was a studio apartment with a kitchen, very tiny. But I tried to be always with people. But there were also bad times. Sometimes I thought “now I am going to go to sleep and tomorrow I’ll see things differently. But that did not happen. Soon everything got complicated, and numbers didn’t add up. Then, there came the bank statements and loneliness … I no longer had any health either.”*

Source [2]: Otero-López, J.M.; Villardefrancos, E. *Adicción a la compra, materialismo y satisfacción con la vida*. Granada, Spain, 2009. ISBN 978-84-9915-092-5999333.
